# The Outcomes of Endovascular Aneurysm Repair in Japan in 2017: A Report from the Japanese Committee for Stentgraft Management

**DOI:** 10.3400/avd.ar.20-00162

**Published:** 2021-03-25

**Authors:** Katsuyuki Hoshina, Kimihiro Komori, Hiraku Kumamaru, Hideyuki Shimizu

**Affiliations:** 1 Department of Vascular Surgery, Graduate School of Medicine, The University of Tokyo, Tokyo, Japan; 2 Division of Vascular Surgery, Department of Surgery, Nagoya University Graduate School of Medicine, Nagoya, Aichi, Japan; 3 Department of Healthcare Quality Assessment, School of Public Health, Graduate School of Medicine, The University of Tokyo, Tokyo, Japan; 4 Department of Cardiovascular Surgery, Keio University, Tokyo, Japan

**Keywords:** endovascular aneurysm repair, abdominal aortic aneurysm, rupture, annual report, Japanese Committee for Stentgraft Management

## Introduction

Endovascular aortic repair (EVAR) was first approved in Japan a decade ago, and approximately 10,000 EVAR procedures are performed annually.^1)^ The short-term outcomes of EVAR in Japan were reported to be acceptable; however, several issues were revealed. Half of the cases violated the instructions for use, and a quarter of post-EVAR aneurysm sacs dilated by more than 5 mm in 5 years.^2)^ In addition, new devices have emerged, generating a learning curve for their use. Therefore, updating the real-world EVAR data from Japan will serve as a database for physicians to refer to during preoperative sizing or when considering procedural steps.

This report demonstrated the number of EVAR procedures and the mortality and complication rates in 2017 in Japan using data from the Japanese Committee for Stentgraft Management (JACSM) nationwide registry, which includes outcome data for nearly all stent grafts shipped to Japan.^2,3)^

## Materials and Methods

### Database, exclusion criteria, and groups

The JACSM nationwide registry, including its foundation, structure, and quality control, has been previously described.^2,3)^ The JACSM, established in December 2006, is composed of 10 societies related to endovascular treatment and determined the practical standards for institutions and for practicing and supervising surgeons. Participating institutions are obligated to report data on EVAR and thoracic endovascular aortic repair (TEVAR), using a web-based case-registry form (http://www.stentgraft.jp/).

**Figure 1:**
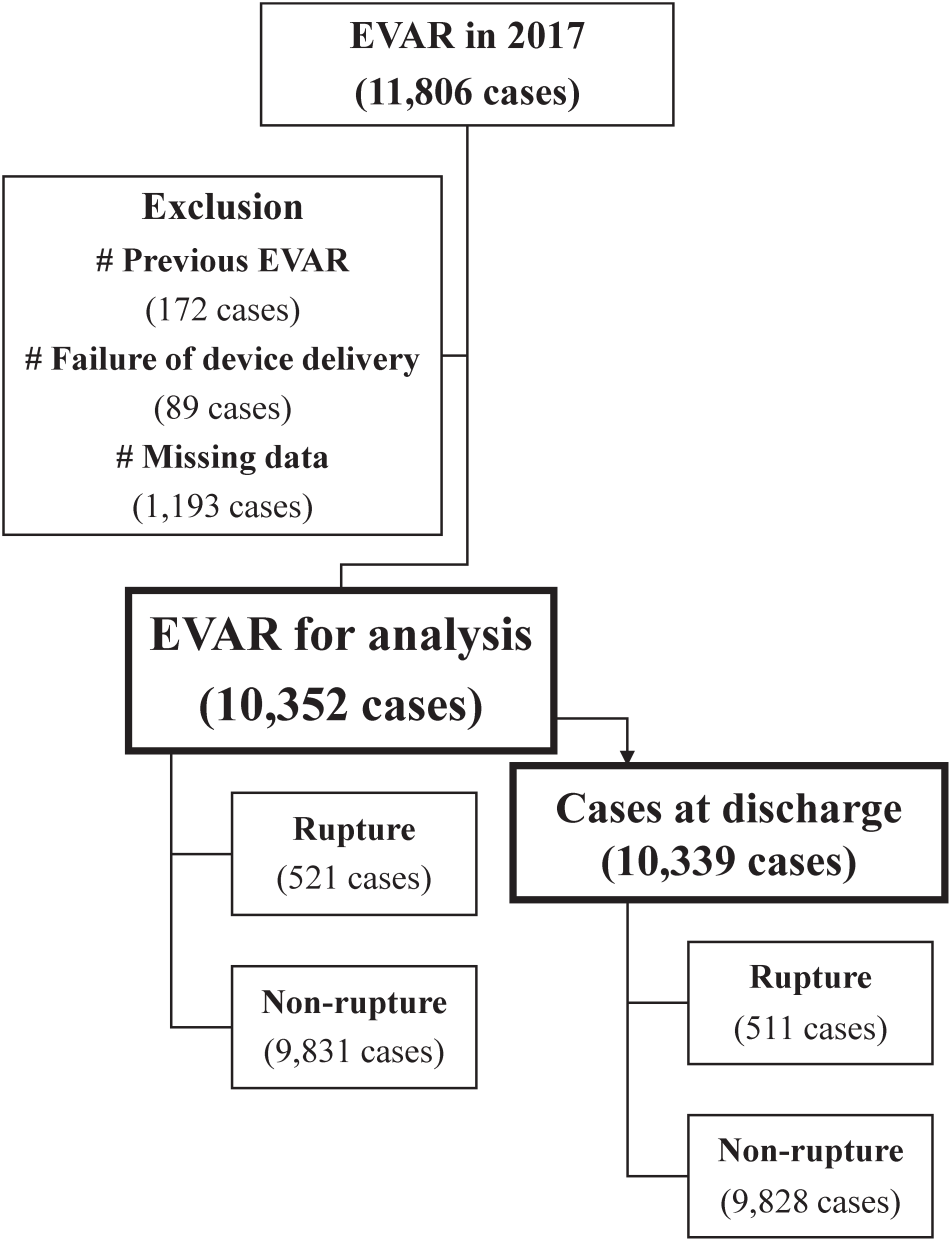
Fig. 1 A flow chart of patient selection from the total patient pool.

Among 11,806 patients who underwent EVAR in 2017, 10,352 patients were analyzed after exclusion based on previous EVAR, failure of delivery, and missing data. The data of 10,339 patients were available at hospital discharge (511 and 9,828 patients in the rupture and non-rupture groups, respectively). Mortality, adverse events, including renal insufficiency and endoleaks, were observed in each group (**Fig. 1**).

This registry was conducted according to the principles of the Declaration of Helsinki, the International Conference on Harmonization, and Good Clinical Practice guidelines. The use of registry data was approved by the Institutional Review Board of the University of Tokyo Hospital (approval number: 2019306NI).

### Type of data collected

Data regarding patient age, sex, aneurysm rupture, device usage, lesion for treatment, dissection, symptoms, pathogenesis, comorbidities, renal function, anatomy of the proximal and distal fixation, and preoperative aneurysm diameter were collected from the hospital database. Patient comorbidities included respiratory disorders, home oxygen therapy, hypertension (with medication), cerebrovascular disease, hemodialysis, coronary artery disease, diabetes mellitus, and a hostile abdomen. Renal function was determined using serum creatinine levels and the estimated glomerular filtration rate (eGFR).

### Outcomes

The intraoperative rates of mortality, vascular injury, rupture, and endoleak were reported. Postoperative mortality and adverse events (migration, stenosis/occlusion, vascular injury, blood transfusion, infection, thromboembolism, renal insufficiency, cerebrovascular damage, paraplegia, rupture, wound trouble, and additional surgery) upon discharge from the hospital were also reported.

Categorical variables are presented as numbers and percentages, and continuous variables are presented as means±standard deviations.

## Results

### Patient demographics

The mean patient age was 76.7±8.5 years. There were 1,920 females (18.5%), and the proportion differed between the rupture group (21.5%) and the non-rupture group (4.2%). EVAR was performed for a ruptured abdominal aortic aneurysm (AAA) in 5.0% of patients. The off-label use of the main body only or the leg only devices was 3.0% and 1.8%, respectively. Regarding etiology, most patients were diagnosed with degenerative (98.0%) and non-dissection (96.9%) conditions. Symptoms were present in 11.1% and more frequently in the rupture group (91.7%). Renal function was worse in the rupture group (eGFR: 47.5±22.2) than in the non-rupture group (eGFR: 57.7±19.6).

The mean aortic aneurysm diameter was 51.6±11.7 mm and was larger on average in the rupture group (66.5±17.3 mm). Regarding the landing zones, approximately 30 mm was secured in all groups at both the proximal and distal sites (**Table 1**).

**Table 1:** Table 1 Patient demographics

	Total		Ruptured		Non-ruptured	
Number of cases	10352		521		9831	
Preoperative data	Cases	Rate (%)	Cases	Rate (%)	Cases	Rate (%)
Female	1920	18.5%	112	21.5%	409	4.2%
Age						
(mean±SD)	76.7±8.5		76.7±9.5		76.7±8.4	
<65	708	6.8%	52	10.0%	656	6.7%
65–74	3022	29.2%	159	30.5%	2863	29.1%
75–84	4850	46.9%	180	34.5%	4670	47.5%
85≤	1772	17.1%	130	25.0%	1642	16.7%
Rupture						
Rupture with enteric fistula	21	0.2%				
Rupture without enteric fistula	500	4.8%				
Non-rupture	9831	95.0%				
Device usage						
Main body+leg	9707	93.8%				
Main body only	309	3.0%				
Leg only	186	1.8%				
Others	150	1.4%				
Lesion for treatment						
Abdominal aorta	7278	70.3%	381	73.1%	6897	70.2%
Abdominal aorta-iliac artery	3074	29.7%	140	26.9%	2934	29.8%
Dissection						
Dissection	322	3.1%	15	2.9%	307	3.1%
Non-dissection	10030	96.9%	506	97.1%	9524	96.9%
Symptom						
Symptomatic	1151	11.1%	478	91.7%	673	6.8%
Asymptomatic	9201	88.9%	43	8.3%	9158	93.2%
Pathogenesis						
Degenerative	10147	98.0%	488	93.7%	9659	98.3%
Inflammation	80	0.8%	7	1.3%	73	0.7%
Aortitis	5	0.0%	0	0.0%	5	0.1%
Infection	62	0.6%	18	3.5%	44	0.4%
Connective tissue disorders	6	0.1%	0	0.0%	6	0.1%
Others	52	0.5%	8	1.5%	44	0.4%
Comorbidities						
Respiratory disorder	1608	15.5%	88	16.9%	1520	15.5%
Home oxygen therapy	66	0.6%	5	1.0%	61	0.6%
Hypertension	6906	66.7%	340	65.3%	6566	66.8%
Cerebrovascular disease	1306	12.6%	70	13.4%	1236	12.6%
Hemodialysis	452	4.4%	18	3.5%	434	4.4%
Coronary artery disease	2238	21.6%	62	11.9%	2176	22.1%
Diabetes mellitus	1482	14.3%	57	10.9%	1425	14.5%
Hostile abdomen	968	9.4%	49	9.4%	919	9.3%
Renal function						
Creatinine (mean±SD) (mg/dL)	1.1±0.7		1.4±0.9		1.1±0.7	
eGFR (mean±SD)	57.2±19.8		47.5±22.2		57.7±19.6	
Proximal fixation						
Diameter (mean±SD) (mm)	21.9±3.9		22.4±4.1		21.9±3.9	
Length (mean±SD) (mm)	33.4±18.0		29.1±18.3		33.6±18.0	
Aneurysm diameter (mean±SD) (mm)	51.6±11.7		66.5±17.3		50.8±10.8	
Distal landing zone (right)						
Common iliac artery	7585	73.3%	390	74.9%	7195	73.2%
External iliac artery	2348	22.7%	104	20.0%	2244	22.8%
Others	419	4.0%	27	5.2%	392	4.0%
Diameter (mean±SD) (mm)	13.1±3.9		13.4±4.5		13.1±3.9	
Length (mean±SD) (mm)	36.8±16.9		33.9±14.4		36.9±17.0	
Distal landing zone (left)						
Common iliac artery	8255	79.7%	411	78.9%	7844	79.8%
External iliac artery	1643	15.9%	76	14.6%	1567	15.9%
Others	454	4.4%	34	6.5%	420	4.3%
Diameter (mean±SD) (mm)	13.3±3.7		13.5±4.1		13.3±3.7	
Length (mean±SD) (mm)	39.8±17.7		39.6±17.2		40.0±17.7	

SD: standard deviation; eGFR: estimated glomerular filtration rate

### Intraoperative data

Intraoperative death occurred in 13 patients (0.1%), 10 of whom were in the rupture group. The rate of intraoperative vascular injury was 1.7%, the rupture rate was 0.1%, and additional procedures were required in 15.5% of cases. Intraoperative endoleak occurred in 566 patients (**Table 2**).

**Table 2:** Table 2 Intraoperative data

	Total		Ruptured		Non-ruptured	
Number of cases	10352		521		9831	
Intraoperative data	Cases	Rate (%)	Cases	Rate (%)	Cases	Rate (%)
Anesthesia						
General	9582	92.6%	485	93.1%	9097	92.5%
Epidural	61	0.6%	0	0.0%	61	0.6%
Local	640	6.2%	33	6.3%	607	6.2%
Others	69	0.7%	3	0.6%	66	0.7%
Endoleaks						
Type 1	516	5.0%	25	4.8%	491	5.0%
Type 2	1274	12.3%	43	8.3%	1231	12.5%
Type 3	90	0.9%	5	1.0%	85	0.9%
Type 4	1421	13.7%	45	8.6%	1376	14.0%
Additional procedures	1602	15.5%	93	17.9%	1509	15.3%
Vascular injury	177	1.7%	12	2.3%	165	1.7%
Aneurysm rupture	13	0.1%	7	1.3%	6	0.1%
Intraoperative death	13	0.1%	10	1.9%	3	0.0%

### Postoperative data

The overall in-hospital mortality rate was 1.2%, with 12.5% of deaths occurring in the rupture group and 0.6% in the non-rupture group. The duration of postoperative hospitalization was 10.0±9.6 days in the non-ruptured group and long in the rupture group (21.3±19.3 days). Renal insufficiency occurred in 3.2% of all patients, and 11.2% of these patients were in the rupture group. Blood transfusion was required in 33.3% and 3.9% of patients in the rupture and non-rupture groups, respectively.

The number of types 1 and 3 endoleaks at hospital discharge was 144 (1.3%) and 58 (0.5%), respectively. The rate of type 2 endoleak was 12.7%, which was similar to the intraoperative rate (12.3%) (**Table 3**).

**Table 3:** Table 3 Postoperative data

	Total		Ruptured		Non-ruptured	
Number of cases	10339		511		9828	
Data at hospital discharge	Cases	Rate (%)	Cases	Rate (%)	Cases	Rate (%)
Duration of hospitalization after the operation						
Days (mean±SD)	10.5±10.6		21.3±19.3		10.0±9.68	
Complications						
Migration	16/9136	0.1%	3/433	0.6%	13/8703	0.1%
Stenosis/Occlusion	93/9138	1.0%	3/433	0.6%	90/8705	1.0%
Vascular injury	45/9137	0.4%	0/433	0.0%	45/8704	0.5%
Blood transfusion	552	5.3%	170	33.3%	382	3.9%
Infection	72	0.7%	25	4.9%	47	0.5%
Thromboembolism	76	0.7%	7	1.4%	69	0.7%
Renal insufficiency	329	3.2%	57	11.2%	272	2.8%
Cerebrovascular damage	43	0.4%	7	1.4%	36	0.4%
Paraplegia	31	0.3%	7	1.4%	24	0.2%
Aneurysm rupture	8	0.1%	5	1.0%	3	0.0%
Wound trouble	99	1.0%	7	1.4%	92	0.9%
Additional surgery	102	1.0%	25	4.9%	77	0.8%
Endoleaks						
Type 1	144	1.3%	8	1.5%	136	1.3%
Type 2	1319	12.7%	44	8.6%	1275	12.9%
Type 3	58	0.5%	4	0.7%	54	0.5%
Type 4	117	1.1%	6	1.1%	111	1.1%
In-hospital death	127	1.2%	64	12.5%	63	0.6%
Aneurysm diameter (mean±SD) (mm)	50.8±11.6		63.2±17.4		50.1±10.8	

SD: standard deviation

### Causes of death

Intraoperative rupture, hemorrhage, and systemic circulatory failure were the most common causes of intraoperative death, accounting for six and two patients in the rupture and non-rupture groups, respectively. During hospitalization, infection or sepsis was the most common cause of death in the non-rupture group (n=11). The next most common causes were pneumonia and respiratory failure (n=10), followed by disseminated intravascular coagulation and multiple organ failure (n=8), arrhythmia, low output syndrome, and heart failure (n=7). Multiple thromboembolisms occurred in five cases, and vascular-related complications, including thoracic aortic aneurysm rupture and aortic dissection, occurred in four cases (**Table 4**).

**Table 4:** Table 4 Causes of death

Causes of death	Rupture	Non-rupture
[During the operation]		
Rupture/Hemorrhage/Systemic circulatory failure	6	2
Acute coronary syndrome	3	0
Acute myocardial infarction	1	0
Enteric necrosis	0	1
**TOTAL**	**10**	**3**
[In-hospital]		
Rupture/Hemorrhage/Systemic circulatory failure	29	2
Acute coronary syndrome	4	0
Arrythmia/Low output syndrome/Heart failure	1	7
Multiple thromboembolism	0	5
Cerebrovascular damage	2	3
Pneumonia/Respiratory failure	6	10
Liver failure	0	1
Renal failure	0	2
Intestinal necrosis/Enterocolitis	5	3
Vascular-related events (TAA rupture, TAD)	0	4
Infection/Sepsis	8	11
DIC/MOF	6	8
Cancer	0	4
Others (sudden death, unknown)	3	3
**TOTAL**	**64**	**63**

TAA: thoracic aortic dissection; TAD: thoracic aortic dissection; DIC: disseminated intravascular coagulation; MOF: multiple organ failure

## Discussion

The JACSM registry began in July 2006 after the approval of the stent graft device in Japan, and data input and storage have been transferred from the JACSM database to the National Clinical Database since January 2016. The analysis with the data through 2015 was published previously.^2)^ The 2016 annual data were reported on the JACSM website, and the committee decided to publish data annually henceforth.

In this study, patients were divided into the rupture and non-rupture groups because of the high mortality and morbidities of ruptured AAA cases. The backgrounds of these groups were too different to match; therefore, we did not compare the outcomes of these groups statistically. It will be necessary to perform a similar study in groups that can be matched so that statistical analyses can be used to confirm our observations.

The mortality of ruptured AAAs varied widely among previous studies, possibly due to selection bias between open surgery and EVAR. A meta-analysis comprised of 8,201 patients who underwent EVAR revealed an in-hospital mortality rate of 30%,^4)^ and the 30-day mortality reported by the NSQIP (National Surgical Quality Improvement Program) database between 2005 and 2007 was 25%.^5)^ A lower mortality rate was reported in a group of high-volume centers (21.2%)^6)^ and in a risk-stratified analysis.^7)^ Although our study used more recent registry data, the mortality rate of 14.2% was considered low, particularly in the rupture group (5%). This could potentially be accounted for if operators only select patients whose anatomy was feasible for EVAR. The outcomes of EVAR for ruptured AAAs were considered acceptable in the current EVAR situation.

The mortality of the non-rupture group was 0.6%, which was lower than the rate between 2006 and 2015 (1.0%).^2)^ Technological advancements in the available devices and improved technical skills likely contributed to the low mortality rate.

The proportion of females was high in the rupture group, which could correlate with higher aneurysm rupture and expansion rates in women compared with that in men.^8,9)^ Although there were other differences between the rupture and non-rupture groups, including decreased renal function, larger aneurysm diameter, and more symptoms in the rupture group, these results were reasonable and expected.

The presence of endoleaks was different intraoperatively than at hospital discharge. The rate of type 1 endoleak decreased at discharge. Although type 1 endoleak should be treated intraoperatively if possible, minor leakage, such as a sleeve leak could be expected to thrombose after the operation.

The duration of postoperative hospitalization was similar in both groups and considered long. In EVAR trial 1, the average length of postoperative stay was 6.9 days.^10)^ The longer stay might be derived from a difference in the health insurance system in each country. In the future, the duration can potentially be shortened in our country by using percutaneous EVAR or intraoperative evaluation with cone-beam computed tomography.

The main causes of death were respiratory and cardiovascular events. The rate of preoperative morbidities potentially affects the causes of death. Surveillance and careful perioperative treatment for comorbidities are likely to be important for the improvement of EVAR outcomes.

The mortality and morbidities of EVAR were acceptable in both rupture and non-rupture groups. The outcomes of the non-rupture group improved compared with that of the previous report with data from 2006 to 2015, possibly due to improvements in devices and operators’ skills.

## Notes

*The JACSM collaborators are listed in the Acknowledgements.
